# A New Immunoassay for Detecting All Subtypes of Shiga Toxins Produced by Shiga Toxin-Producing *E*. *coli* in Ground Beef

**DOI:** 10.1371/journal.pone.0148092

**Published:** 2016-01-29

**Authors:** Xiaohua He, Qiulian Kong, Stephanie Patfield, Craig Skinner, Reuven Rasooly

**Affiliations:** 1 Western Regional Research Center, U.S. Department of Agriculture, Agricultural Research Service, Albany, California, United States of America; 2 Shanghai Shuneng Irradiation Technology Co., Ltd, Shanghai Academy of Agricultural Sciences, Shanghai, China; The Pennsylvania State University, UNITED STATES

## Abstract

**Background:**

Shiga toxin (Stx) is a common virulence factor of all Shiga toxin producing *E*. *coli* (STEC) that cause a wide spectrum of disease, including hemorrhagic colitis and hemolytic uremic syndrome (HUS). Although several commercial kits are available for detection of Stx produced by STEC, none of them are capable of recognizing all subtypes of Stxs, which include three subtypes of Stx1 and seven subtypes of Stx2.

**Methods and Findings:**

New monoclonal and polyclonal antibodies against Stx1 and Stx2 were developed. A universal sandwich ELISA capable of detecting all known subtypes of Stx1 and Stx2 was established using a pool of newly developed antibodies. To precisely monitor the sensitivity of the assay for each subtype of Stxs, recombinant toxoids were created and used as standards in ELISAs. Because of the high affinity of the antibodies incorporated, the ELISA assay is highly sensitive with a limit of detection for the different subtypes of Stx1a and Stx2a between 10 and 50 pg/mL in phosphate buffered saline (PBS). The assay was also able to identify STEC based on the production of Stxs using the supernatants of culture fluids or even single colonies on agar plates without lengthy enrichment in liquid medium. When applied to ground beef samples, this newly developed ELISA was capable of distinguishing beef samples spiked with a single bacterial cell.

**Conclusions:**

A highly sensitive and universal assay for all subtypes of Stx1 and Stx2 was developed. It has significantly improved upon the current technologies by avoiding false negative results due to the narrow detection range of the assay. The assay developed in this study can be useful for prompt detection of new and emerging serotypes and screening ground beef samples for contamination of STEC at an early stage in the food supply chain, thus avoiding the need for possible recall.

## Introduction

Shiga toxin-producing *Escerichia coli* (STEC) are a group of bacteria responsible for approximately 100,000 cases of illness and 3,000 hospitalizations each year in the United States alone. Eight percent of patients hospitalized from STEC infections develop hemolytic uremic syndrome (HUS), a life-threatening disease [1]. Before 2012, the strategy for diagnosis of clinical samples mainly relied on biochemical markers, which was based on the unique sorbitol negative fermentation and ß- D-glucuronidase-positive properties of the O157 strains [2, 3]. Therefore, the most frequently identified STEC associated with reported outbreaks was E. coli O157:H7 serotype. However, as more laboratories start to use non serotype based assays, more illness and outbreaks linked to non-O157 STEC serotypes are uncovered. In a report published in 2012, six non-O157 serotypes, O26, O45, O103, O111, O121 and O145, were revealed to be responsible for 113,000 illness annually in the United States alone, almost double the amount of illness caused by O157 [4]. Other sera-groups, including the highly virulent *E*. *coli* O104:H4, have also caused large outbreaks of diarrhea and HUS [5]. It is clear that non serotype-based methods for detection of all STEC strains are needed.

One common trait of all STEC strains is the ability to produce Shiga toxin (Stx), which is one of the most important virulence factors associated with human illness. Therefore, a method relying on this common trait of all STEC and not individual serotype identification would be a better strategy for diagnosis purposes. PCR assays specific for *stx* genes have been commonly used for the identification of STEC. These assays are sensitive and specific, however, their target is the *stx* gene sequence, not the toxin itself. In addition, false-positive and false-negative results are obtained, occasionally, due to the presence of cryptic target gene sequences such as defective *stx* genes or PCR inhibitors present in the samples. A more reliable method would be to use the production of Stx as a marker for viable STEC. Vero cell assay and mouse bioassay have been the gold standards for detection of Stxs, but these assays are time-consuming, labor intensive, and require special facilities and trained personnel. Furthermore, these assays are non-specific, and a subsequent antibody-based neutralization assay is required to confirm the presence of the Stx. The enzyme-linked immunosorbent assay (ELISA) has been broadly used for the detection and quantification of proteins, it provides several benefits, including small sample volumes and hence lesser amounts of reagents; easy to adapt to high throughput applications, and the ability to wash away nonspecifically bound materials for measuring specific analytes within complex matrices. Furthermore, all reagents and equipment needed by ELISA are available in most laboratories. However, only a few ELISA kits for Stxs are commercially available. Those that are available are normally overwhelmingly expensive and not effective at detecting all serotpes of STEC [6].

There are two types of Stx, Stx1 (almost identical to Shiga toxin produced by *Shigella dysenteriae* type 1) and the immunologically distinct type Stx2. According to a recent sequence-based protocol for subtyping Stxs, three subtypes of Stx1 (Stx1a, Stx1c, and Stx1d) and seven subtypes of Stx2 (Stx2a through Stx2g) have been classified. Among each subtype, different numbers of variants (ranging from 1 to 21) have been reported [7]. Although these numerous toxins are similar structurally, with an active *N*-glycosidase A-subunit associated with 5 identical B-subunits, their broad genetic variations present a challenge for the development of universal assays capable of detecting all subtypes of these toxins. Previously, we reported a polyclonal antibody-based ELISA that was able to detect seven subtypes of Stx2 produced by STEC strains in overnight culture media [8]. Building on the success of that ELISA, herein we introduce our new Stx1 and Stx2 antibodies; report the development of a new assay that detects not only all subtypes of Stx2, but also all subtypes of Stx1; and demonstrate the improvement of the new assay compared to previous assays on the sensitivity of detection for Stxs. Since cattle have been identified as one of the major reservoirs of STEC and a large number of STEC outbreaks have been associated with eating undercooked ground beef, therefore, we further validate the new assay for the detection of STEC spiked in ground beef based on the production of Stx and demonstrate that the new assay is capable of detecting Stx in enriched ground beef culture spiked with a single STEC cell, suggesting the feasibility of using this ELISA to screen STEC in ground beef.

## Materials and Methods

### Ethics statement

All procedures with animals were carried out according to institutional guidelines for husbandry approved by the Institutional Animal Care and Use Committee of the U.S. Department of Agriculture, Western Regional Research Center (USDA IACUC). Specific procedures and protocols for antibody production were reviewed and approved by the USDA IACUC (Protocol# 09-J-10). Mice were euthanized using rapid cervical dislocation to minimize suffering.

### Bacterial strains

Bacterial strains used for generation of Stx toxoids are shown in Table 1. Twenty four Stx-negative and positive bacterial strains containing *stx1a*, *1c*, *1d* and *stx2a* through *stx2g* genes used to assess the ability of the ELISA for detecting subtypes of Stxs are listed in Table 2. These isolates came from our bacterial strain collection housed in the Produce Safety and Microbiology unit at USDA, ARS, Western Regional Research Center. Stock bacterial strains were maintained in 20% glycerol and frozen at -80°C. Fresh bacterial cultures were produced by inoculating frozen stock cultures onto tryptic soy agar (TSA) plates and incubating the plates overnight at 37°C.

**Table 1:** Strains used to generate toxoids.

Toxoid generated	Source strain	Other name	Serotype	*stx* genotype	Origin	Reference
Stx1aE167Q	RM2084	EDL933	O157:H7	*stx1a*, *stx2a*	Meat	[9]
Stx1cE167Q	FF6		O113:H4	*stx1c*, *stx2b*	ND^a^	[10]
Stx1dE167Q	RM13149		ND^a^	*stx1d*	ND^a^	[11]
Stx2aE167Q	RM10638		ND^a^	*stx2a*	Cow	[12]
Stx2bE167Q	RM7005	EH250	O188:H12	*stx2b*	Clinical	[13]
Stx2cE167Q	RM10058		O157:H7	*stx2c*	Bird	[12]
Stx2dE167Q	RM8013		ND^a^	*stx2d*	Cow	[12]
Stx2eE167Q	RM7110	S1191	O139:NM	*stx2e*	Pig	[14]
Stx2fE167Q	RM7007	T4/97	O128:H2	*stx2f*	Feral pigeon	[15]
Stx2gE167Q	RM10468		ND^a^	*stx2g*	Cow	[12]

^a^Not determined.

**Table 2:** Detection of Stx in culture supernatants of STEC strains by ELISA.

Strain	Serotype	*stx* genotype	s/n	SD	s/n^a^
RM13506	O45	*stx1a*	1622	21	++++
RM13508	O103	*stx1a*	1728	46	++++
RM9882	O103	*stx1a*	1595	19	++++
AA1	O174:H8	*stx1c*, *stx2b*	1915	15	++++
FF6	O113:H4	*stx1c*, *stx2b*	1720	74	++++
RM13149	nd	*stx1d*	1305	10	++++
II9	O41:H26	*stx1d*	772	6	+++
RM8082	O121	*stx1d*	847	69	+++
RM10638	O157:H7	*stx2a*	218	4	+++
RM1913	O157:H7	*stx2a*	222	1	+++
RM13504	O121	*stx2a*	215	3	+++
RM7005	O118:H12	*stx2b*	118	1	+++
RM10058	O157:H7	*stx2c*	155	1	+++
MC654	O145:H28	*stx1a*, *stx2c*	221	1	+++
RM8013	nd	*stx2d*	139	1	+++
RM7958	O113	*stx1a*, *stx2d*	142	1	+++
RM7110	O139:NM	*stx2e*	9	1	+
RM7988	nd	*stx2e*	160	1	+++
TW05622	O138	*stx2e*	23	1	++
RM7007	O128:H2	*stx2f*	138	1	+++
CC3	O128:H2	*stx2f*	137	0	+++
RM10468	nd	*stx2g*	155	4	+++
RM4876	O157:H7	-	1	0	-
ATCC25922	O6	-	1	0	-

^a^ELISA signal to noise ratio (s/n): s/n < 2, -; 10 ~100, ++; 100 ~1000, +++; >1000, ++++

### Production of recombinant Stx toxoids

Recombinant toxoids of Stx1a, Stx2a, and Stx2e have been described, previously [11, 16, 17]. Stx1c, Stx1d, Stx2b, Stx2c, Stx2d, Stx2f, and Stx2g toxoids were produced in this study as described for the toxoids of Stx1a and Stx2a [11, 16]. Briefly, genomic DNA was isolated from *stx*-expressing bacterial strains listed in Table 1. A point mutation (replacing glutamic acid at position 167 of Stx to glutamine) was introduced into *stx* genes by PCR. The full-length *stx* gene with a point mutation was cloned into the pQE-T7-2 vector (Qiagen, Valencia, CA) and expressed in BL21(DE3)pLysS competent cells (Promega, Madison, WI). Stx toxoids were purified using an affinity column coupled with an appropriate antibody [16]. The purity of toxoids was analyzed by polyacrylamide gel electrophoresis (PAGE) as described, previously [16]. Briefly, 1 μg of toxoid was separated by SDS-PAGE using a 4–12% NuPAGE Novex Bis-Tris mini protein gel (Invitrogen, Carlsbad, CA). Gels were stained with Simply Blue Safe Stain (Invitrogen) for protein visualization.

### Antibody production

Stx2b monoclonal antibodies were produced using protocols described, previously [18]. Briefly, a construct containing Stx2b B-subunit DNA sequence was developed using the pTrcHis2 TOPO vector (Invitrogen). His-tagged Stx2b B-subunit was then expressed in TOP10 cells (Invitrogen) and purified using a Ni-NTA affinity column (Qiagen, Valencia, CA). Purified Stx2b B-subunit (5 μg/mouse) in Sigma adjuvant system (Sigma-Aldrich) was used as an immunogen and injected into mice at two-week intervals for a total of three injections. Two weeks after the third injection, mice were boosted with 1 μg/mouse of Stx2b B-subunit in sterile PBS. Four days later, mice were sacrificed by rapid cervical dislocation, spleens were excised aseptically, and splenocytes were harvested. SP2/0 myeloma cell and splenocyte cell fusions were achieved using a polyethylene glycol-based protocol. Clonal hybridoma lines were then achieved with three rounds of cloning by limited dilution, regrowth, and screening.

Production of Stx1a polyclonal antibody was performed by Pacific Immunology Corp (Ramona, CA) as described for Stx2 polyclonal antibody production, previously [8]. Briefly, rabbits were injected with 300 μg of Stx1a (E167Q) toxoid at 3-week intervals for a total of four injections. Bleeds were collected and evaluated for their binding to Stx1a toxoid. Antibodies were purified using Protein-A affinity column (Pierce, Rockfield, IL). Protein concentration was determined with the BCA Protein Assay Kit (Pierce).

### Enzyme-linked immunosorbent assay (ELISA)

The universal sandwich ELISA was performed as described previously with slight modification [16]. Briefly, plates were coated with a mixture of capture antibodies including Stx1-2 [11], Stx2-5 [16], Stx2b-1 (this study), Stx2e-2 [17] and Stx2f-1 [19] (100 μL, 1 μg/mL of each antibody in PBS) and incubated overnight at 4°C, followed by washing and blocking with 300 μL/well blocking solution (5% milk in TBST) for 1 hour at 37°C. After brief washing, the indicated purified toxin/toxoid, bacterial culture supernatant or colonies were then added (100 μL) and incubated at 37°C for 1 hour. The plates were washed six times with TBST, then a mixture of Stx1 and Stx2 polyclonal antibodies (100 ng/mL each, diluted in 100 μL of blocking solution) was added and incubated for 1 hour at 37°C. The plates were washed a further six times, 100 μL/well of 10 ng/mL goat anti-rabbit IgG-HRP (GAR-HRP, Promega, Madison, WI) in blocking solution was added. After incubation for 1 hour at 37°C, the plates were washed a final six times and developed using Pico chemiluminescent substrate (Thermo Scientific). Luminescence was measured using a Victor 3 plate reader (Perkin-Elmer, Shelton, CT). All sandwich ELISAs were conducted at least three times for confirmation. Limit of detection (LOD) was calculated by extrapolating ng/mL of Stxs from the background luminescence plus 3 standard deviations of the background.

### Western blot analysis

Western blots were conducted as previously described [16]. Pure toxin/toxoid were denatured at 72°C for 10 minutes in 1x NuPage LDS loading buffer, and then separated on a 4%–12% NuPAGE Novex Bis-Tris mini gel (Invitrogen). The proteins were transferred to a PVDF membrane (pore size, 0.45 μm), blocked with 2% ECL Prime blocking agent (GE Healthcare) in PBST, and washed with PBST (3x). Monoclonal or polyclonal antibodies were diluted to 1 μg/mL in blocking solution and incubated with the blots for 1 hour at room temperature. After washing (3x) in PBST, the blots were incubated with GAR-HRP or goat anti-mouse HRP (GAM-HRP) antibodies (Promega) at 5 ng/mL for 1 hour at RT. The blots were developed using Lumigen TMA-6 (Lumigen) substrate and visualized using a FluorChem HD2 (Alpha Innotech). All western blots were analyzed at least three times.

### Preparation of bacterial samples for ELISA

For colony ELISA, bacterial strains were inoculated onto TSA plates and incubated overnight at 37°C. Single colonies (~ 1mm in diameter) were picked with a pipette tip and re-suspended in a micro tube containing 100 μL triptic soy broth (TSB), 100 μL Buffered Protein Extraction Reagent (B-PER in phosphate buffer, Pierce Biotechnology, Rockford, IL) and mitomycin C (50 ng/mL). After incubation at 37°C for 1 hour, cell debris were removed by centrifugation and the supernatant was used as the Stx source for ELISA analysis.

To prepare *E*. *coli* culture supernatant for ELISA, a colony of each bacterial strain was inoculated to a flask containing 25 mL TSB and 50 ng/mL mitomycin C and cultured for 18 hours at 42°C. For Stx1 detection, overnight broth culture was incubated with an equal volume of B-PER solution for another hour at 37°C to elicit toxins from bacterial cells (Stx1 is often associated with cells). Following centrifugation at 13,000 x g for 10 min at 4°C, bacterial supernatants were collected and filtered through a 0.2 μm filter for use in ELISA.

### Detection of Stx in ground beef inoculated with STEC

Ground beef marked 90% lean was purchased at a local supermarket and packed into small single use bags and stored at -80°C before use. For enrichment cultures containing ground beef (Table 3), an overnight axenic culture was serially diluted into buffered peptone water, and an appropriate dilution was used to inoculate 25 g of ground beef. Actual inoculum levels were later determined via spread plating 0.1 mL of diluted cultures onto TSA and incubating overnight at 37°C. Inoculated ground beef samples were enriched at 42°C for 18 hours in flasks containing 75 mL TSB, 50 ng/mL mitomycin C. For beef samples inoculated with Stx1-producing strains, overnight enrichment cultures were incubated with an equal volume of B-PER solution for another hour at 37°C. Following centrifugation at 13,000 x g for 10 min at 4°C, bacterial supernatants were collected and filtered through a 0.2 μm filter for use in ELISA.

**Table 3:** Detection of Stx in group beef samples spiked with STEC strains.

Bacterial strain	Predicted cfu	Actual cfu	ELISA s/n	SD	s/n^a^
RM13506 (stx1a)	1	2	349	64	+++
	5	2	391	74	+++
	10	9	443	80	+++
RM13508 (stx1a)	1	0	2	0	+
	5	4	334	59	+++
	10	14	343	58	+++
RM10638 (stx2a)	1	1	2	0	+
	5	2	2	0	+
	10	2.5	691	88	+++
RM1913 (stx2a)	1	0.5	3	0	+
	5	3	949	100	+++
	10	5.5	1039	136	++++
ATCC25922 (- stx)	100	100	1	0	-
TSB medium	0	0	1	0	-

^a^ELISA signal to noise ratio (s/n): s/n < 2, -; 10 ~100, ++; 100 ~1000, +++; >1000, ++++

## Results

### Preparation of standards for different subtypes of Stx1 and Stx2

**Fig 1:**
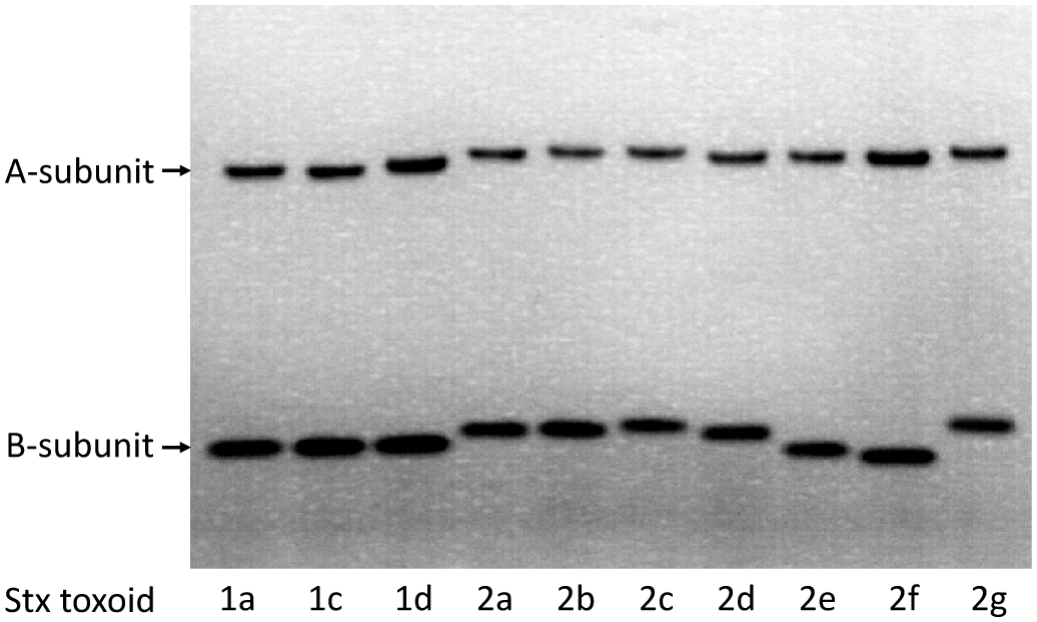
Exhibition of recombinant Stx toxoids following SDS-PAGE. Each lane was loaded with 1 μg of toxoid purified by affinity column. The gel was stained with Simply Blue Safe Stain (Invitrogen). The 32 kDa A-subunit and 7-kDa B-subunit of toxoids are indicated by arrows.

Genomic DNAs were isolated from STEC reference strains expressing Stx1a, 1c, 1d and Stx2a, 2b, 2c, 2d, 2e, 2f, and 2g (Table 1). Recombinant toxoids of each Stx subtype were generated by changing each subtype’s mature toxin active site, glutamic acid (E) at position 167 of the A subunit to glutamine (Q) as described in Materials and Methods. After being expressed in *E*. *coli*, recombinant toxoids from cell lysates were purified using antibody-coupled affinity columns. The antibody used for purifying Stx1a, 1c, and 1d, was our previously developed mAb Stx1-1 [11], mAb Stx2-2 [16] was used for purifying Stx2a, 2c, 2d, and 2g, mAb Stx2e-3 [17] was used for Stx2b and 2e, and mAb Stx2f-4 [19] was used for Stx2f. The purity of each toxoid after affinity purification was assessed following SDS-PAGE by Simply Blue staining. Two protein bands were observed with molecular weights of 32 kDa and 7 kDa, corresponding to the sizes of the A and B subunits and no contaminating proteins were visible in any purified subtypes of Stx1 and Stx2 toxoid preparations (Fig 1). These recombinant toxoid preparations were used as standards in ELISA tests.

### Development of a universal ELISA for detection of all subtypes of Stx1 and Stx2

**Fig 2:**
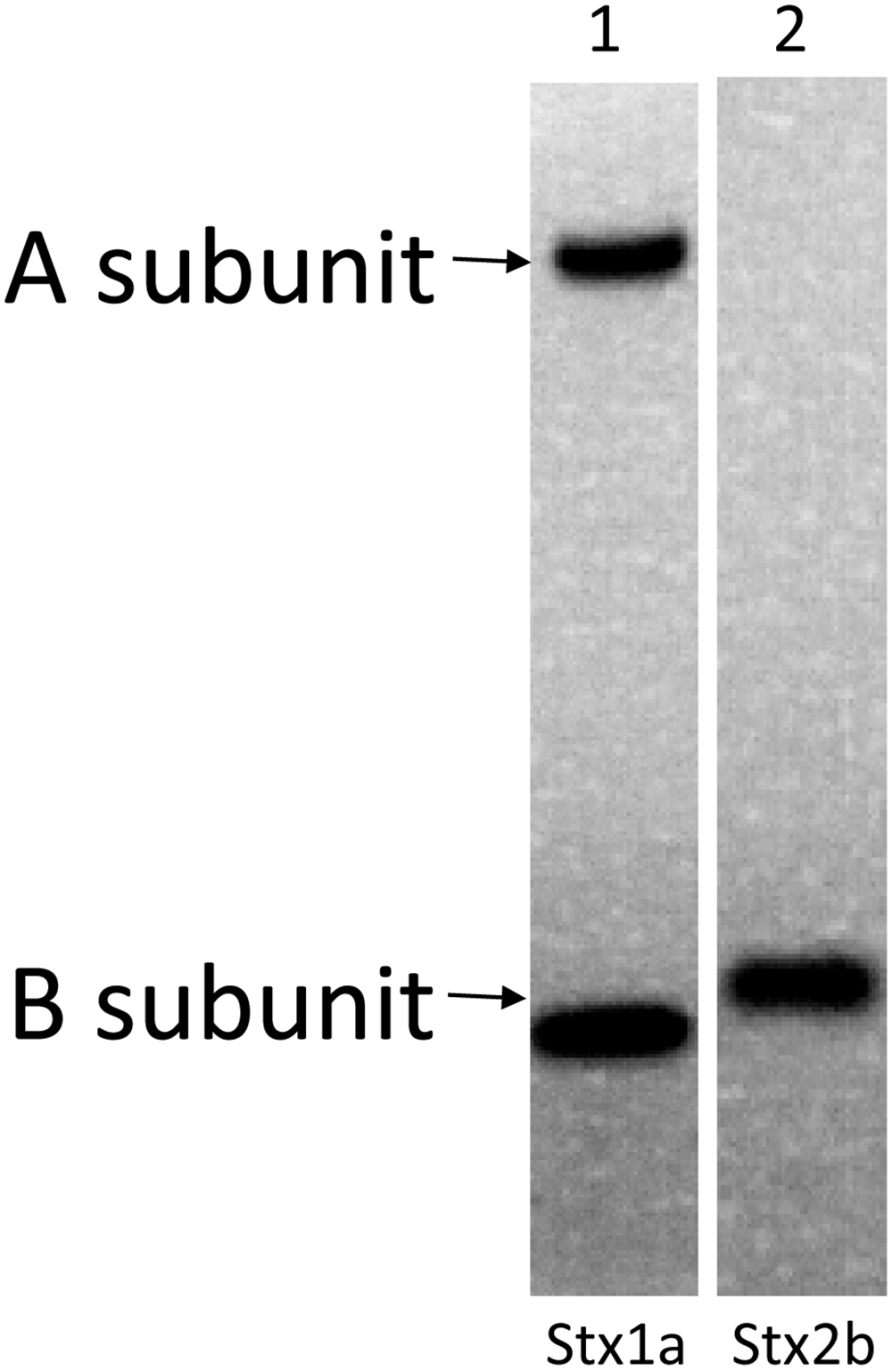
Analysis of antibody binding to Stxs by Western blot. Lane 1 was loaded with pure Stx1a (0.5 μg) and lane 2 was loaded with 10 X concentrated overnight bacterial cultural supernatant containing Stx2b (10 μL). Proteins were separated by SDS-PAGE, transferred to PVDF membranes, and probed with rabbit polyclonal antibody against Stx1a (lane 1), and mAb Stx2b-1 (lane 2), respectively. The positions of the A- and B-subunit of the Stx1a and Stx2b are indicated at the left side of the panel.

**Fig 3:**
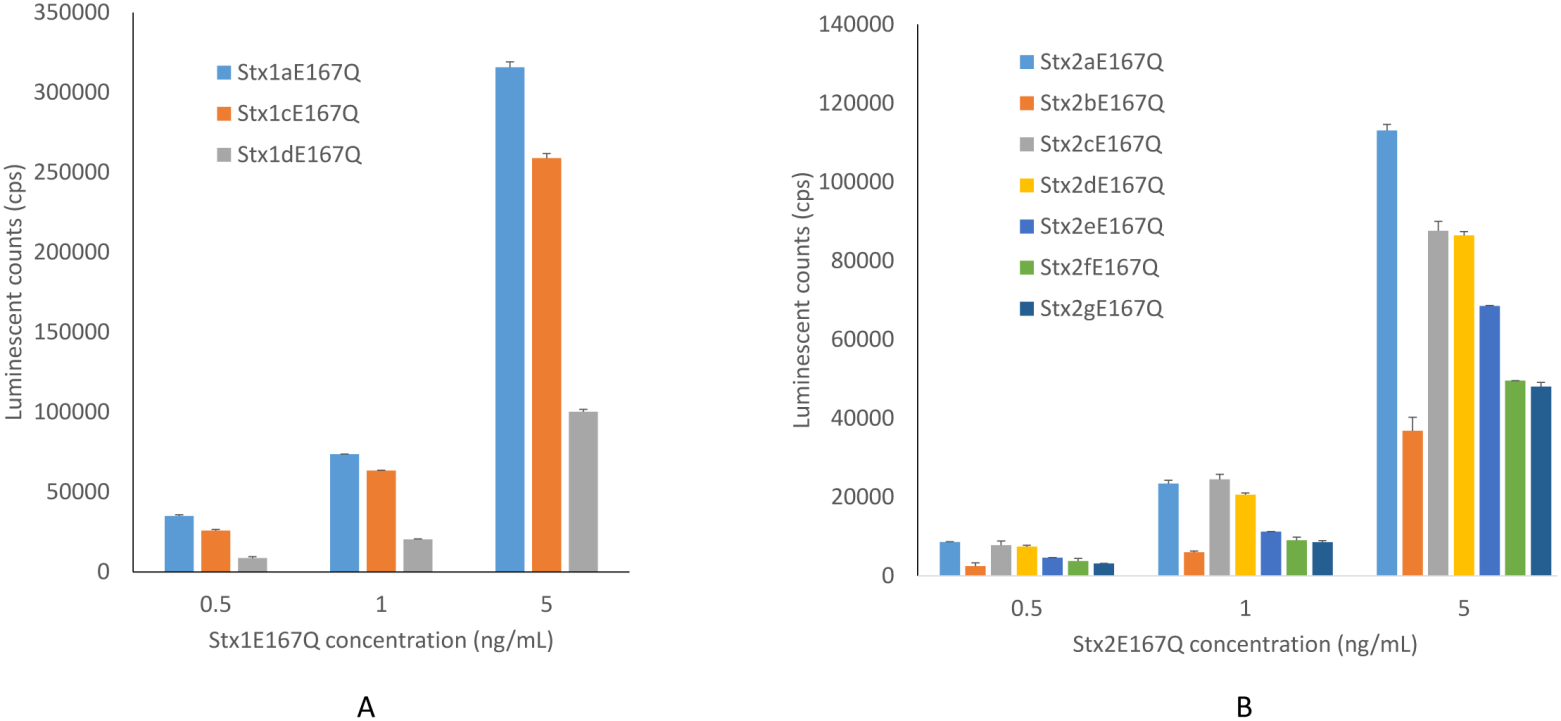
Detection of subtypes of Stx1 and Stx2 toxoids using the universal sandwich ELISA. A mixture of mAbs including mAbs Stx1-2, Stx2-5, Stx2b-1, Stx2e-2, and Stx2f-1 was used for capture (1 μg/mL each) and a mixture of Stx1 and Stx2 pAbs was used for detection (100 ng/mL each), the goat anti-rabbit IgG-HRP was used as a secondary antibody (10 ng/mL). The data shown represent the mean plus SD of three replicates from one representative experiment. Three individual experiments were performed.

To establish a highly sensitive immunoassay that is capable of detecting all subtypes of both Stx1 and Stx2, antibodies are the most crucial components. We have reported the development of mAbs against Stx1a, 1c, 1d [11], Stx2a, 2c, 2d, 2g [16], Stx2e and Stx2f [19], and polyclonal antibody (pAb) against Stx2 [8]. But high affinity mAbs against Stx2b and polyclonal antibody with broad reactivity to Stx1 are still not available. In this study, new mAbs against Stx2b and pAb against Stx1 were generated (see Materials and Methods). Among four mAbs developed, one mAb, named Stx2b-1, was selected for use in the ELISA. Stx2b-1 possesses a kappa light chain and an IgG1 type heavy chain. Western blot analysis indicates that this mAb recognizes the Stx2b produced by *E*. *coli* and is B-subunit specific (Fig 2, lane 2). The Stx1 pAb developed in rabbit binds to both A and B subunits of the native Stx1 (Fig 2, lane 1). A sandwich ELISA was then assembled using a mAb cocktail containing mAbs Stx1-2, Stx2-5, Stx2b-1, Stx2e-2, and Stx2f-1 for capture and a mixture of Stx1 and Stx2 polyclonal antibodies for detection. For quantification purposes, purified recombinant toxoids of Stx subtypes prepared in this study were used as standards. Fig 3 demonstrates that all subtypes of Stx1 (A) and Stx2 (B) toxoids were detected by this ELISA. Linear standard curve with R^2^ between 0.99 and 1 was obtained for each subtype of Stxs when using toxin concentrations between 0.05 to 5 ng/mL. The LODs for Stx1 subtypes, 1a, 1c were 10 pg/mL and for Stx1d was 50 pg/mL; for Stx2 subtypes, 2a to 2g were between 25 and 50 pg/mL.

### Detection of Stxs generated in STEC enrichment cultures

To investigate the ability of this ELISA for detection of native Stxs produced by STEC strains, 22 STEC strains carrying different *stx* genes were first incubated overnight with mitomycin C to induce the toxin expression, and culture supernatant of each strain was then collected for ELISA analysis. For Stx1-producing strains, bacterial cultures were subsequently treated with B-PER, a protein extraction reagent, following mitomycin C induction to release cell-associated Stx1 (Stx1 is known to be more cell-associated compared with Stx2, which is mostly soluble). Table 2 exhibits ELISA results for Stx levels expressed in the form of signal to noise ratio (s/n). It indicates that the sandwich ELISA is able to detect all subtypes of Stx1 and Stx2 produced by STEC strains. The signal to noise ratio of the ELISA for Stx1 ranges from 776 to 1925 among Stx1a, 1c, and 1d, much higher than that for the seven Stx2 subtypes (Stx2a to 2g), which ranges from 8 to 222. It was noticeable that the ELISA signals from two Stx2e strains, RM7110 and TW05622, were significantly lower than other strains.

To investigate the feasibility of using this ELISA to detect STEC without a lengthy enrichment step, single colonies of bacterial strains (about 1 mm in diameter) collected from TSA plate were directly tested for the presence of Stx. Our results indicate that 16 out of 18 STEC strains were identifiable based on the production of Stx. Medium to high level of toxins (s/n > 10) were detected in 13 strains and low levels of toxins (s/n between 2 and 10) were detected in three strains. The assay failed to detect Stx in two strains containing the *stx2e* genes. The ELISA did not cross-react with two *stx*-negative control strains.

### Sensitivity of the ELISA for detection of Stx in STEC-inoculated ground beef

To test the suitability and sensitivity of the ELISA for detection of STEC in ground beef based on the production of Stx, strains expressing Stx1a and Stx2a (two strains each) were used to inoculate ground beef following the USDA FSIS protocol with slight modifications. Table 3 exhibits the semi-quantitative results from ELISA for the production of Stx1 and Stx2 by bacteria in ground beef after overnight growth. The signal to noise ratio (s/n) for Stx was found to be at the limit of detection boundary level (2 to 3) in three out of four samples originally inoculated with a single cell in 25 grams of ground beef. However, the s/n ratio increased to more than 334 in most samples (three out of four) inoculated with 5 cells and the s/n ratio was equal or above 343 in all samples inoculated with 10 cells in 25 grams of ground beef.

## Discussion

STEC is one of the well-known foodborne pathogens. Because of its high pathogenicity in humans, these pathogens have rapidly become a major concern. To identify outbreaks, potential sources of ongoing transmission and prevent further transmission from the sources, reliable methods for detection of STEC are crucial. For this purpose, a variety of methods has evolved [20]. Stx as a common trait of all STEC, was considered one of the most reliable targets for diagnosis of STEC, and several commercial ELISAs have been developed [21, 22]. While a few studies have evaluated the ability of Stx ELISA kits for detecting different subtypes of Stx1 and Stx2, it is difficult to compare these results because most of the studies used crude Stx samples such as bacterial culture supernatant or cell lysates and the actual sensitivity of the test was not known. Purification of Stxs from wild type pathogenic bacteria is considerably cumbersome, requiring a large volume of culture and multiple chromatography steps. It’s extremely difficult to purify subtypes such as Stx2b, Stx2e and Stx2f because the amounts of these toxins produced by bacteria are generally very low [23]. Currently, there are no commercial standards for subtypes of Stxs available except for Stx1a and Stx2a. In order to evaluate the ELISA sensitivity for each subtype of Stxs, we generated a set of standards that includes all known subtypes of Stxs. Considering public biosecurity concerns about generating biologically active toxins using recombinant DNA technology, we constructed non-toxic recombinant toxoids by converting the conserved glutamic acid at position 167 of the Stxs to glutamine, then used them as standards in our assays for quantification purposes. As shown in Fig 1, these standards are uniformly pure without noticeable contamination on stained SDS-PAGE. The A- and B-subunit migration on the protein gel are very similar for all subtypes of Stx1, including Stx1a, 1c and 1d. But slight differences in A- and B-subunit migration were observed among the 7 subtypes of Stx2. The B-subunits of Stx2e and Stx2f migrated significantly faster than the other 5 subtypes. The predicted B-subunit molecular weights of all 7 Stx2 subtypes based on amino acid sequences are very similar. It’s not clear why the Stx2e and Stx2f B-subunit appears smaller on the gel.

Currently, no universal immunoassay for all subtypes of Stx1 and Stx2 is available. The Meridian Premier EHEC assay recommended by USDA-FSIS for confirmation of the presence of Stxs in the protocol for detection of STEC is one of the most widely used commercial assays in the field, however, it barely detects Stx2b, 2e, and 2g [8]. Our previously reported polyclonal antibody-based ELISA detected all seven subtypes of Stx2, but not Stx1 and the sensitivity of the assay to Stx2b, Stx2e and Stx2f was poor [8]. The inability of an assay to detect some subtypes of Stxs has become a concern because this could cause misleading diagnostic results, resulting in the risk of a disease-causing STEC outbreak. To develop an ELISA with broader specificity and improved sensitivity, we developed new high affinity antibodies with specificities that complement our existing mAbs. To increase the assay’s recognition for Stx2b, mAbs were generated using His-tagged Stx2b B-subunit as an immunogen. At the same time, a pool of mAbs were screened for their binding to Stxs. Finally, a mixture of mAbs, including mAbs Stx1-2, Stx2-5, Stx2e-2, Stx2f-1, and a newly developed mAb, Stx2b-1, were selected for use as a capturer, and a mixture of a Stx2 pAb [8] and the Stx1 pAb developed in this study were selected to serve as a detector. By incorporating these antibodies, a new ELISA was assembled. This ELISA was demonstrated to detect all subtypes of Stx1 and Stx2 (Fig 3A and 3B) and the limit of detection for different subtypes were between 10 and 50 pg/mL.

In order to examine the ability of the new assay to detect Stx produced by culture-enriched STECs, 22 STEC strains were tested. As indicated in Table 2, this ELISA was able to detect all subtypes of Stx1 and Stx2 in strains that contain *stx* genes. But we noticed that the ELISA signals from Stx1-producing bacterial samples were much higher than that from Stx2-producing bacterial samples. This could be because the strains carrying *stx1* genes produce more toxins in general, but it is also possible that the antibodies used in the ELISA have higher affinity to subtypes of Stx1 than to subtypes of Stx2. The second hypothesis is supported by the ELISA data using the pure toxins, which also shows that the ELISA signal to noise ratio is higher for Stx1 than for Stx2 although the same amount of toxin was applied.

When we compared this ELISA with assays reported previously [8], we found that the sensitivity of this ELISA is improved 1.3 to 12-fold over the polyclonal antibody-based ELISA and 2.8 to 78-fold over the Meridian Premier EHEC for Stx2a to 2g produced by the same strains. The improvement in assay sensitivity for Stx2b, Stx2e and Stx2g was particularly dramatic compared with the Meridian Premier EHEC. We conclude the improvement is mainly due to the incorporation of high affinity antibodies in the new ELISA. We were also interested in evaluating the ability of our assay to identify STEC using single colonies grown on agar plates as a testing material without liquid enrichment steps. We tested 18 strains (two colonies from each strain) and found that this ELISA was able to recognize 16 out of 18 STEC strains. The two that failed were Stx2e-producing strains and known to produce very low amounts of Stx2e even in overnight enrichment culture, which was also observed by other researchers [23]. These results suggest that strains expressing very low amount of Stxs may go undetected using the single colony approach, although it could save a significant amount of assay time.

Cattle are the principal reservoir of STEC [24]. Ground beef was found to be linked to 336 severe foodborne outbreaks between 1998 and 2010 and over 100 cases were caused by STEC [25]. Therefore, we sought to validate the ability of our ELISA to detect Stx in ground beef. Ground beef homogenates (25 g in 75 mL TSB with 50 ng/mL mitomycin C) spiked with 1–10 bacterial cells were directly subjected to ELISA after overnight enrichment. Positive results were obtained even in samples inoculated with a single bacterial cell, indicating that incubation of contaminated ground beef samples at 42°C overnight substantially elevated the growth of STEC and production of Stxs. No toxin was detected in samples inoculated with a non STEC strain (100 cfu/25 g), suggesting that it is feasible to use Stx production as a marker for the presence of viable STEC in ground beef.

## Conclusions

Previously, we made recombinant toxoids for Stx1a, Stx2a and Stx2e. In this study, we made recombinant toxoids for Stx1c, Stx1d, Stx2b, Stx2c, Stx2d, Stx2f, and Stx2g. These toxoids are valuable reagents for ELISAs and antibody production. Using new antibodies against Stx1 and Stx2b developed in this study, together with our previously developed antibodies, a unique ELISA that detects all subtypes of Stx1 and Stx2 was established. The ELISA was highly sensitive and the LOD for different subtypes of Stxs ranges between 10 and 50 pg/mL based on our new toxoid standards. Twenty-two STEC strains expressing three subtypes of Stx1 and seven subtypes of Stx2 were readily identified after overnight enrichment in liquid medium with no false positive or negative results observed. Except for the two Stx2e-producing strains, this ELISA also recognized all STEC strains using single colonies on agar plates as targets without further enrichment in liquid medium. Most importantly, this ELISA detected as few as one STEC cell in 25 grams of ground beef after overnight incubation. Although only four STEC strains were tested and the sensitivity of detection for different strains varied widely, all samples inoculated at this level were detectable. When the inoculation level was increased to 5 cells or more in 25 grams of beef, the results became more reliable. This study suggests that the new ELISA has the potential to reduce STEC-associated outbreaks by reducing failures of detecting STEC strains that produce rare subtypes of Stxs. It is also useful for meat processing plants to perform in-house testing of products prior to sale, therefore reducing the frequency of product recalls and enhancing public health.
